# Periodic pattern formation during embryonic development

**DOI:** 10.1042/BST20230197

**Published:** 2024-01-30

**Authors:** Zoe R. Sudderick, James D. Glover

**Affiliations:** The Roslin Institute & R(D)SVS, University of Edinburgh, Edinburgh, U.K.

**Keywords:** developmental biology, embryogenesis, periodic patterns, turing systems

## Abstract

During embryonic development many organs and structures require the formation of series of repeating elements known as periodic patterns. Ranging from the digits of the limb to the feathers of the avian skin, the correct formation of these embryonic patterns is essential for the future form and function of these tissues. However, the mechanisms that produce these patterns are not fully understood due to the existence of several modes of pattern generation which often differ between organs and species. Here, we review the current state of the field and provide a perspective on future approaches to studying this fundamental process of embryonic development.

## Introduction

**Figure 1.**
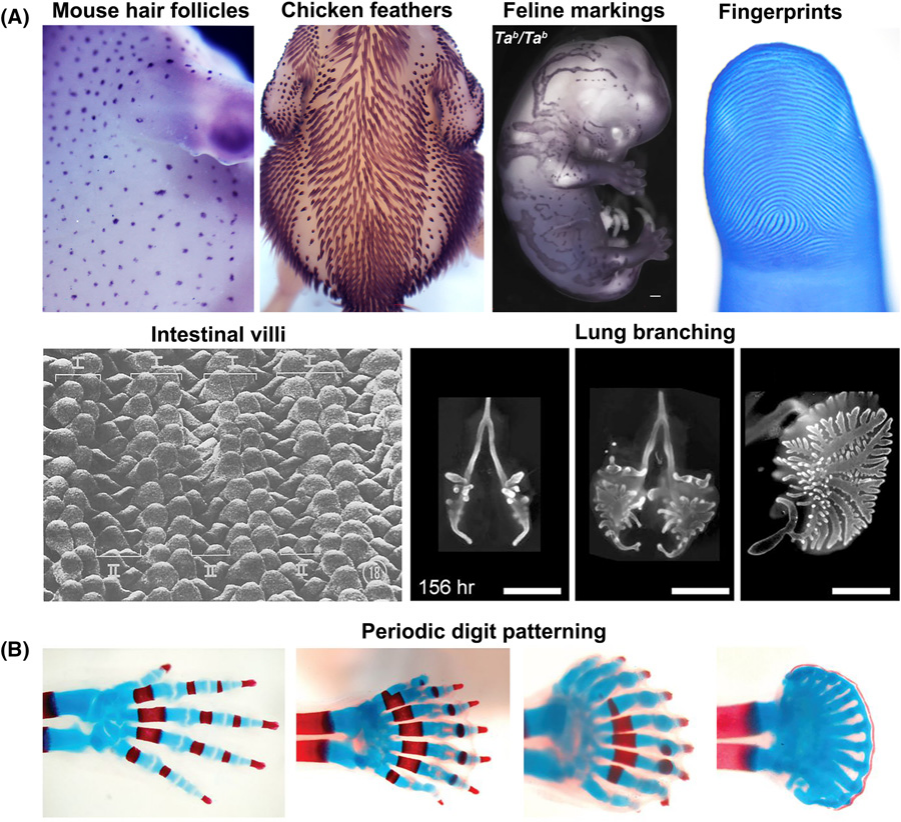
Periodic pattern formation during embryonic development. (**A**) Examples of embryonic periodic patterns. Top panel from left to right: Mouse primary hair follicle pattern at E13.5 visualised by *Dkk4 in situ* hybridisation; *in situ* hybridisation detecting *SHH* in the developing feathers of an E13 chicken embryo; Feline colouration pattern forming during embryonic development visualised by *Dkk4 in situ* hybridisation; Fingerprint ridge pattern in a human embryonic digit at week 15 estimated gestational age. Bottom panel from left to right: Regularly patterned villi in E19 chicken embryo intestine; Periodic branching events visualised through E-cadherin staining of embryonic chicken lungs between E6 and E7.5. (**B**) The digits of the limb form through a periodic patterning process. Altering the levels of Hox genes leads to increased digit number and reduced interdigital spacing (wavelength). Feather image used with permission of William Ho. Cat image from Kaelin et al. [63]. Fingerprint image from Glover et al. [9]. Villi image from Grey [106]; reprinted with permission from John Wiley and Sons. Chicken lung images from Tzou et al. [107]. Mouse limb images from Sheth et al. [2]; reprinted with permission from the American Association for the Advancement of Science.

The generation of structure from an initially homogeneous state, such as how an entire organism is developed from a single fertilised egg, is an enduring and fascinating scientific question [1]. During embryonic development, the formation of spatially distinct repeating elements is essential for the correct function of many tissues and organs. These arrangements are called periodic patterns and are usually observed as spots or stripes. Forming autonomously from an initially homogenous state, embryonic periodic patterns include the digits of the limb [2,3], the appendages of the skin (including hair follicles [4–6], feathers [7,8] and fingerprints[9]), and the villi of the intestine [10,11] (Figure 1). Although the final structure and function of these patterns differ vastly between organs and species, the tissues in which they form are similar, consisting of two layers, a tightly packed epithelium which sits above a more loosely arranged mesenchyme. It is the interaction and interplay between these two tissue layers that defines the periodic pattern and ultimately, the final morphology of an organ [12]. Here, we provide an overview of the mechanisms involved in the generation of these periodic patterns during embryogenesis and discuss the latest research approaches which may provide insight into the study of these events moving forward.

## Modes of pattern formation

**Figure 2.**
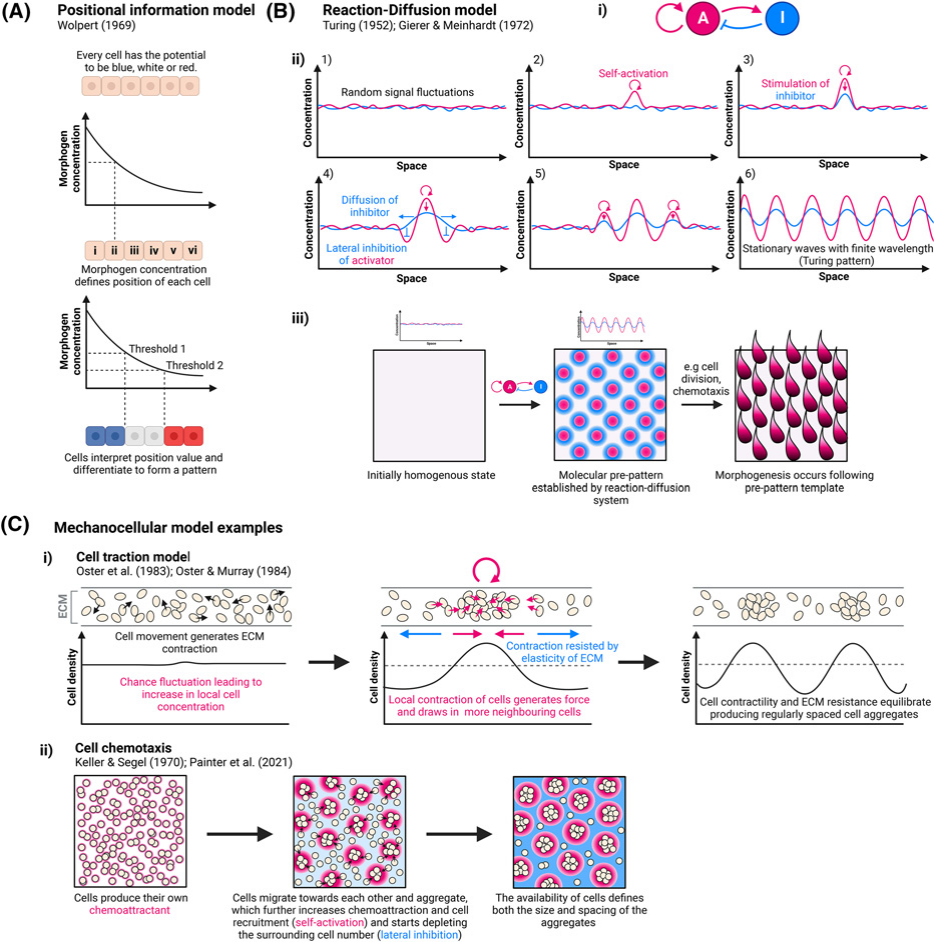
Modes of embryonic pattern formation. (**A**) Schematic representation of Wolpert's positional information (French flag) model. Populations of cells have the developmental potential to become several different fates (depicted as blue, white or red). Exposure of a morphogen gradient across the populations results in cells gaining a unique positional value based on the concentration of morphogen they experience. This positional information is then interpreted by the cell and it differentiates into a blue, white or red fate in accordance with a predetermined genetic programme. (**B**) Features of RD models. (i) Schematic representation of the interactions between the two components of a basic RD system — an activator (A) and inhibitor (I). The activator promotes the production of both its own inhibitor and itself (self-activation). (ii) Even within an apparently homogenous system molecular fluctuations exist across the patterning space. As cells experience a slightly higher concentration of activator this will be enhanced further through self-activation. Because the activator also stimulates inhibitor production, the concentration of the inhibitor also increases. Because the inhibitor diffuses faster than the activator this leads to lateral inhibition in the surrounding cells as activator levels fail to grow. The faster diffusion of the inhibitor also means that at the activator peak, inhibitor levels never accumulate high enough to supress the activator. However, new peaks of activator can form past the regions of lateral inhibition and the whole process of self-activation and lateral inhibition is repeated until a regular array of activator peaks and valleys forms across the patterning field. (iii) From an initially homogenous state a molecular periodic pre-pattern is produced by a RD system. This then provides a template for cells to follow and undergo morphogenesis. (**C**) Examples of MC models. (i) In a cell traction model migrating cells within a layer of ECM generate traction. A random fluctuation leading to an increased number of cells in a specific area leads to an increase in local cell contraction which draws in more cells (self-activation). This contraction is resisted by the elasticity of the ECM limiting the number of cells that can be drawn in through contraction (lateral inhibition). The balance between these events produces evenly spaced aggregates. (ii) In a chemotaxis model, all cells within a system produce their own chemoattractant. As cells migrate towards each other they aggregate which creates a larger source of chemoattractant to recruit additional cells (self-activation). As more cells are recruited this depletes the number of cells in the surrounding area (lateral inhibition) which both limits the expansion of the aggregate (size) and also the position where another aggregate (spacing) can form. Images created with BioRender.com.

Perhaps the most well-known mechanism to produce periodic patterns is from the mathematician, wartime code breaker, and father of modern computing, Alan Turing. In his seminal 1952 work [13], Turing proposed that chemical substances, through their diffusion and reaction with each other, are sufficient to amplify intrinsic heterogeneities in a tissue to produce a periodic pattern from an initially near homogenous state, thus ‘breaking symmetry’. Turing coined the term ‘morphogens’ for these chemicals to convey the idea that these are ‘form inducers’ [13]. In these Turing reaction–diffusion (RD) systems, two or more morphogens reach an equilibrium in alternating concentrations based on their interactions with each other and their differential rates of diffusion. However, Turing's ideas were largely forgotten until decades later, partly due to the popularity of Lewis Wolpert's positional information [14,15], or French flag, model where cells acquire a positional value based on the morphogen concentration they experience (Figure 2). Gierer and Meinhardt [16], initially unaware of Turing's work, expanded the RD model and proposed that periodic patterns could form through a pair of interacting morphogens, termed an ‘activator’ and ‘inhibitor’. In these systems, a slowly diffusing activator stimulates the production of both itself and its own fast-diffusing inhibitor, the overall result of which is short-range activation coupled with long-range inhibition (Figure 2). The interaction and diffusion of these morphogens lay out a chemical ‘pre-pattern’ which is used as a template by cells to undergo morphogenesis (Figure 2). It is only over the last couple of decades that it has been possible to experimentally test whether Turing RD systems can produce embryonic periodic patterns. Turing RD systems have now been implicated in the formation of a diverse array of embryonic periodic patterns including the digits of the limb [3,17], hair follicles [4–6], intestinal villi [11], and palatal rugae [18], as well as playing a role in tooth morphogenesis [19], the left–right patterning of the embryo [20,21], and lung branching [22].

However, in addition to RD systems, it has been demonstrated both *in silico* and *in vivo* that periodic patterns can be produced through cell-based or mechanical models [10,23–32], which we now collectively refer to as mechanocellular (MC) models. The principles of MC models are similar to those of RD systems, and incorporate short-range activation and long-range inhibition to generate a periodic pattern. Rather than chemical morphogens, MC models utilise differences in chemotaxis, cell mobility, or through the generation and response to mechanical strain resulting from the interactions between cells and the extracellular matrix (ECM) [12,27] (Figure 2). Although similar patterns are produced by either patterning mode, in MC systems, unlike RD models where a chemical pre-pattern is set up first for cells to interpret, pattern formation and morphogenesis are indistinguishable [24] (Figure 2).

Despite producing similar patterns, the broad category of patterning mechanism operating in a system can be identified by designing specific experiments [27]. For example, mechanical-based patterning mechanisms are likely not involved if perturbing tissue stiffness does not affect the pattern outcome. However, additional mathematical modelling and more rigorous experimental design are then required to further differentiate between the specific mechanisms within each class [27].

In this mini review, we will mainly focus on periodic patterns generated by RD or MC mechanisms. Our aim is not to be all encompassing, rather we hope to provide an overview of the types of models which can produce these patterns and explore examples of their formation in specific organs and across taxa. It will become increasingly apparent that this is not a one-shoe-fits-all type of problem, with different mechanisms being able to produce the same outcome, and often the same organ may form differently across species. Furthermore, growing evidence suggests it is increasingly likely that a combination of RD and MC patterning modes may co-ordinate to produce the intricate periodic patterns of the embryo.

## Patterns of the skin: from feathers to fingerprints

The skin is the largest organ of the body and depending on the species, its appendages include secretory glands, hairs, feathers, and scales, which serve a multitude of functions, including protection, thermoregulation, communication, and sensory perception [33].

**Figure 3.**
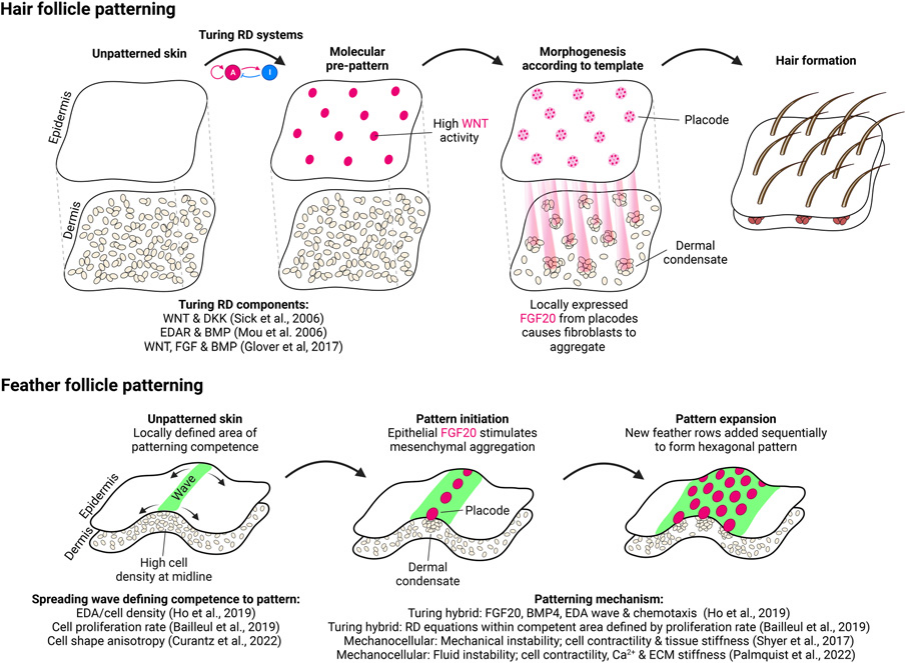
Models of hair follicle and feather patterning. Top: In hair follicle patterning a Turing RD system generates a molecular pre-pattern, consisting of high regions of WNT signalling, which defines sites of future placode formation in the epidermis. FGF20 generated by the placode attracts underlying dermal fibroblasts which then aggregate to form the dermal condensate. The placode and dermal condensate then undergo several subsequent stages of morphogenesis to produce mature hair follicles. Bottom: Feather patterning begins at the midline with the generation of a single row of feathers. As development continues new rows are added sequentially in a wave spreading from the midline to generate a hexagonal pattern in flighted birds. Both MC models, involving mechanical instability, and Turing-like systems incorporating FGF20-mediated chemotaxis, have been proposed to describe the process. Refer to the main text for more details. Images created with BioRender.com.

Primary hair follicle formation in mice begins at E13.5, with each individual hair follicle consisting of an epithelial placode directly associated with a dermal condensate, a structure essential for further hair morphogenesis [34]. Over the next few days, the hair follicles cover the surface of the skin in a regularly spaced periodic pattern of spots (Figure 1) which has been proposed to be driven by Turing-type RD models [4–6]. Initially, models based on two components (acting as an activator and inhibitor) including the WNT and DKK [4], and EDAR and BMP [5] signalling pathways were proposed to generate the primary hair follicle pattern. More recently, we showed that a more complex network of interacting molecules from WNT, FGF, and BMP pathways was capable of producing the periodic pattern [6]. As per the previous studies, we found WNTs are activators whereas BMPs are inhibitors of hair follicle formation. We also demonstrated that a molecular pre-pattern (hallmark of RD systems) is present in the mouse epidermis and provides the template for the local aggregation of mesenchymal cells which form the dermal condensate [6,35,36] (Figure 3). Strikingly, we also discovered that under certain conditions the mesenchyme is capable of patterning without an epidermal pre-pattern template [6]. This ‘mesenchymal self-organisation’ is driven by MC patterning involving TGFβ mediated chemotaxis and demonstrated that both RD and MC patterning modes can be present within a single system. During hair follicle formation, these systems operate in a hierarchical manner where one is subordinated to the other to ensure the fidelity of the pattern. This showcases multiple periodic patterning systems coexisting in a single entity and is something we will encounter, albeit in different guises, in other systems.

In flighted birds, the feathers form during embryogenesis in a neat hexagonal periodic pattern which evolved to aid flight [7,37] (Figure 3). Chickens (Figure 1) and quail retain this layout, but unlike in mouse where the majority of the primary hair follicles form simultaneously, feathers form in a wave beginning as a single row at the midline at E6.5 with new rows being sequentially added over the next 2 days [38].

Like hair, feathers consist of an epithelial placode and associated dermal condensate, requiring both components to produce a mature structure. Over the past couple of decades, some of the molecular events governing feather formation have been elucidated; FGF signalling, including epidermally expressed FGF20, has been shown to be activatory in feather formation [7,39–42], whereas BMP signalling is inhibitory [8,43–45]. However, it was only more recently that the global processes defining the periodic pattern have been explored in greater depth.

A lack of an identifiable pre-pattern led to the proposal that feather formation is initiated by mechanical processes based on the inherent tension generated by the mesenchyme leading to epidermal compression which triggers a β-catenin signalling cascade [29]. Here, feather follicles emerge through mechanical instability, with cellular contractility serving as a short-range activator and tissue stiffness as a long-range inhibitor. Additional evidence to support this came from an *ex vivo* model which suggested that the initiating event in feather formation occurs through contractile mesenchymal cells at the midline aligning the ECM. This further increases its contractility through calcium signalling eventually leading to contractile instability and symmetry breaking into feather primordia [46]. However, neither of these works fully explain how the hexagonal feather arrangement is generated and how patterning spreads as a wave.

Ho and colleagues expanded upon this and proposed that a hybrid patterning system, consisting of an RD system combined with chemotaxis, is capable of producing the hexagonal feather layout. They found that a travelling wave of EDA, the ligand for EDAR, defines the precise location, based on a minimum cell density, in which a periodic pattern can operate to produce a feather row. In permissible regions, FGF20-mediated chemoattraction (activator) facilitates mesenchymal aggregation which leads to the expression of BMP4 (inhibitor) and the compression of the epidermis to enhance further FGF20 expression (self-activation). These authors also did not detect a molecular pre-pattern, demonstrating that although feather and hair follicle formation utilise similar signalling components (WNT, FGF, EDA, BMP) the patterning processes are distinct. Furthermore, a study of feather formation across diverse avian species revealed a universal patterning system also combining elements of RD systems and chemotaxis, whilst additionally incorporating proliferation, mediates the propagation of the patterning wave in different birds [37].

This research on feather formation highlights the complexities of studying periodic pattern formation especially when there is likely an intricate interplay between the mechanical and cell signalling environments driving feather pattern formation, rather than a sole mechanism, which agrees with another recent study exploring the contribution of cell shape anisotropy to feather pattern fidelity [47].

**Figure 4.**
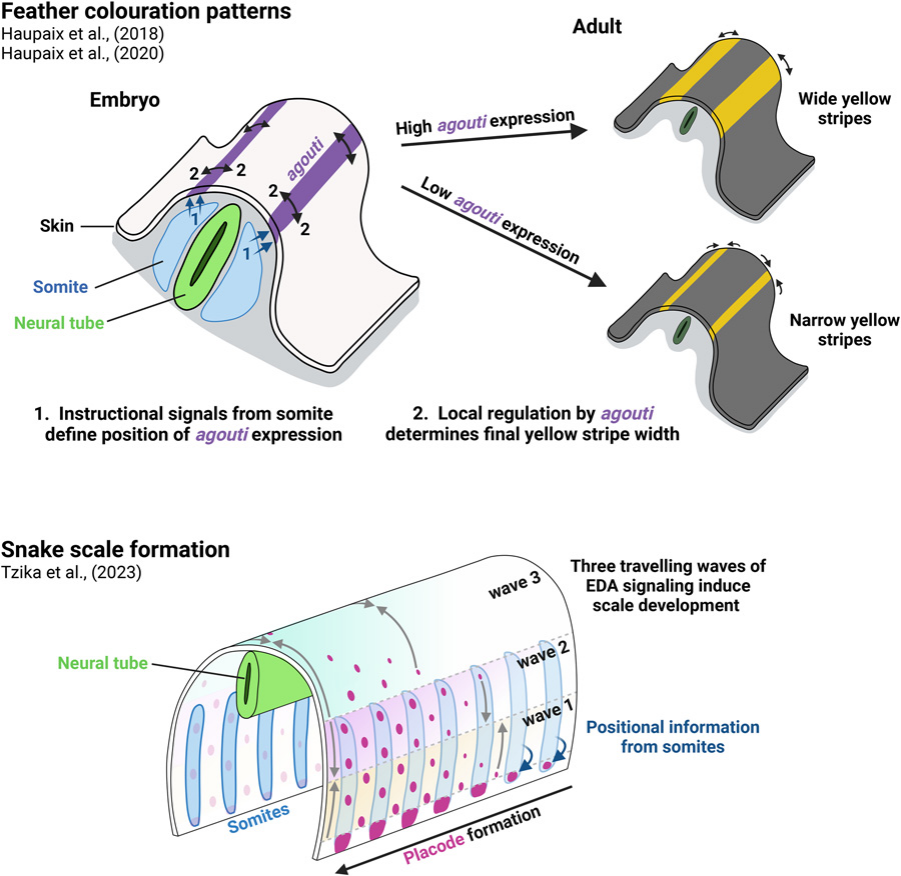
Examples of positional information interacting with patterning mechanisms. Top: Feather colouration patterns in *Galliformes* originate in the embryo. Instructional signals from the somites define the position at which *agouti* expression, and subsequent yellow feather colouration in the adult, occurs. A second patterning system, regulated by *agouti*, then defines the size and spacing of the stripes. Bottom: Snake scale formation occurs through a Turing-like system interacting with three spreading EDA waves that define competence to the pattern. Positional cues from the somites are necessary to define the initial placode location, without which the correct pattern fidelity cannot be generated. Images created with BioRender.com.

Although the patterning modes between mouse hair and avian feathers are distinct, there is conservation of the core signalling components. Indeed, this is actually widespread across taxa. For example, disruption of EDA/EDAR signalling in humans [48], zebrafish [49], mice [50,51], lizards [52], and snakes [53] leads to aberrant ectodermal appendages, affecting the formation of teeth, hair, sweat glands, and scales in these species. Furthermore, two recent studies [53,54] revealed that spreading EDA/EDAR signalling waves, akin to that described for chicken feathers [7], are required for the formation and sequential patterning of periodically arranged scales in zebrafish and snakes (Figure 4) suggesting core patterning mechanisms have evolved to produce these highly specialised patterns. Further demonstration of core patterning components being utilised throughout evolution is the pattern of ectodermal appendages in sharks and turtles. Shark denticles are believed to pattern by a Turing RD system with FGFs and SHH serving as activators and BMP4 acting as an inhibitor [55]. Alternatively, turtle scutes are thought to employ a multi-step patterning process, utilising two sequential RD systems incorporating SHH and FGF4, which follow an initial pre-pattern layout based on somite position [56], with EDAR also playing a role in the patterning process [57].

In the volar skin of humans and other species, rather than hair follicles, a series of evenly spaced epithelial ridges known as dermatoglyphs form which create complex patterns at the digit tips called fingerprints. These fingerprint ridges are molecularly similar to early hair follicles but lack a mesenchymal component, and are hypothesised to form through a Turing-like RD mechanism [9]. In this system, an epidermal pre-pattern based on activatory WNT/EDAR signalling and inhibitory BMP signalling defines local sites of proliferation to drive downgrowth [9]. This patterning system operates as a series of spreading waves initiating from distinct anatomical sites which are influenced by the architecture and signalling environment of the developing digit [9,58]. The timing and interactions of these spreading waves is what determines the final fingerprint pattern type.

## The skin ii: colouration patterns

The colouration patterns of animals such as the stripes of a zebra and spots of a leopard have long been described as being a Turing pattern [59–62], as similar patterns are easily produced by simple RD models, including those described in Turing's original work [13]. However, here we only describe colouration patterns which arise during embryogenesis.

A recent study into feline coat markings [63] provided a molecular context as to how a leopard's spots might indeed form. In this elegant study, the authors demonstrated that the distinctive patterns of domestic cats are produced during embryonic development with a pre-pattern of gene expression preconfiguring sites of epithelial thickness. The WNT inhibitor, *Dkk4* was identified as a key component in this process, with mutations in the gene underpinning the Ticked pattern type. This led to a RD system being proposed to explain the patterning process, in which WNT ligands serve as short-range activators and WNT antagonists act as long-range inhibitors.

Another excellent example exploring the mechanisms defining colour patterns focused on the feather pigmentation patterns across different avian species [64]. Intriguingly, the authors report that the generation of characteristic black and yellow stripes seen in juvenile Galliformes occurs in a two-step process. Firstly, positional cues from the somitic mesoderm define the precise location in the dermis where bands of *agouti* expression, and subsequently yellow stripes, form. Secondly, this pre-pattern is further refined based on the levels of *agouti* expression which temporally determine the pigment type production and thus the width of the stripes (Figure 4). Utilising positional instructions from the somites, prior to the employment of a self-organising mechanism to produce periodicity, ensures directionality and reproducibility of the final pattern by defining the initial elements (longitudinal stripes) [65].

## Teeth and rugae

Both RD-like systems [19,66–68] and MC models [69,70] have been reported to explain various aspects of tooth morphogenesis during embryogenesis. For instance, a RD system with WNT, SHH, and the WNT/BMP inhibitor Sostdc1 serving as the activator, mediator, and inhibitor, respectively, is capable of generating the spacing of mammalian teeth [67]. Turing-like RD systems involving EDAR signalling can also explain how differences in tooth morphology between animals [68,71], particularly molar cusp shape and number, have evolved. More recently, it was also suggested that the differential jaw growth between closely related species of bats directly perturbs the underlying Turing mechanism leading to modulation of the size and spacing of teeth, which likely has enabled these animals to adapt to different dietary requirements [19].

In the developing palate, a series of periodically arranged ridges called rugae form, with a Turing-like system originally consisting of two components, SHH (activator) and FGF (inhibitor) [18] proposed to explain their patterning. Further experimental and computational work, has revealed that additional Turing RD systems utilising up to five components including members of WNT, BMP, SHH, and FGF families across the epithelium and mesenchyme, may drive the final rugae patterning [72,73]. Interestingly, palatal rugae morphology has also been associated with differences in tooth number [74], suggesting that the signalling components driving periodic pattern formation in each system are conserved.

## Intestine

The intestine forms thousands of evenly spaced structures called villi, which increase the surface area and aid nutrient absorption [75] (Figure 1). In the chick gut, a series of folding events occur, including transitioning through a zig-zag state to create the final functional units. The generation of these patterns occurs through confined mechanical strain which leads to directional folding based on the development of the surrounding musculature [10]. In mouse, muscle-induced folding does not occur. Rather, epithelial Hedgehog signalling, interacting with other key developmental signalling pathways including BMP and PDGF, promotes local mesenchymal cell aggregations which undergo further patterning and arrangement in a Turing-like manner leading to villi formation [11,76,77]. A recent preprint has proposed an interesting alternative mechanism for the formation of mouse villi, based on mesenchymal dewetting [78]. Here, a thin layer of PDGFRA^High^ tissue actively acquires fluid-like properties that enable separation from the surrounding PDGFRA^Low^ tissue, leading to the formation of a series of patterned mesenchymal cell aggregates; the size and spacing of which can be modified by modulating the initial number of PDGFRA^High^ cells [78]. Through enhanced cohesion these aggregates round-up and initiate folding in the epithelium above, thereby marking the position of future villus outgrowth [78].

## Limbs

The developing limb forms many patterned structures including the individual digits and joints of the hand. However, these structures have not always been thought of as periodic patterns. Indeed, it was the increased digit number coupled with reduced interdigital spacing and bifurcations, hallmarks of a periodic patterning process, that are present in *Hoxa13/Gli3* mutant mice [2] which provided experimental evidence for this *in vivo* (Figure 1). Prior to this, the specification of digit identities was largely attributed to the concepts of positional information [15], which we briefly discussed earlier (Figure 2), although it should be pointed out that an RD system producing the periodic array of digits was suggested [79,80]. It was only until recently that a Turing-like system consisting of a network of BMP, SOX9, and WNT signalling was shown to be capable of producing the periodic patterning of the digits in mice [3] with a similar model proposed for the patterning of condensations in the catshark pectoral fin [17]. Alternative non-typical Turing RD systems have also been used to model digit formation [27] including Murray and Oster's original mechanical model [81], and further re-evaluation [82] of the *Hoxa14/Gli3* mutant limbs suggested that digits initiate as spots (digit organising centres), likely specified by a Turing system, which then form stripes (digits) through elongation of the limb bud. To add a further layer of complexity, Parada et al. [31] suggest that although a Turing system may drive the periodic patterning of the metacarpals, the digits themselves arise from organising centres of high Activin/pSMAD signalling defined by local mechanical feedback.

During later limb development, Turing-like systems have been used to model the formation of interphalangeal joints [83]. Firstly, models using two separate Turing patterning systems, one which defines dots, and one stripes, were shown *in silico* to be capable of explaining joint number, position, and orientation across various vertebrate species. Secondly, a recent paper [84] combining experimental data and *in silico* modelling suggested that a BMP-based Turing system may be behind these patterning events.

## Other examples of periodic patterning

In the mammalian inner ear, the hair cells and supporting cells in the organ of Corti become periodically arranged through a mechanical patterning system based on global shear and local repulsion forces [85].

During early embryogenesis RD systems have been proposed as an alternative to the clock and wavefront models of somitogenesis [86], and in governing left–right asymmetry through Nodal and Lefty interactions [20,21]. Indeed, the demonstration that during zebrafish embryogenesis the activator Nodal has a lower diffusion rate than the inhibitor Lefty provided biophysical support for the fundamental underpinnings of the classical RD system [13,16].

## Discussion

Many different patterning systems utilise the same suite of signalling pathways including WNT, BMP, FGF, SHH, and EDAR, but are employed in different contexts. This explains why in developmental disorders, such as hypohydrotic ectodermal dysplasia [87] and Robinow syndrome [88], multiple patterning defects are observed together. WNT/β-Catenin signalling is on the whole activatory, as is the case for feathers [7], hair follicles [4,6], fingerprints [9], rugae [72], and feline pigmentation [63]. FGF signalling also appears to be activatory, promoting chemotaxis in several systems [6,7,41,42]. Interestingly, BMP signalling largely serves as an inhibitory influence [5–7,9,11,45,55], often regulating the spacing of patterned elements especially in systems with a high epithelial input such as the skin.

Different mechanisms for embryonic pattern formation have in the past been considered mutually exclusive, and often one type may be dismissed as incapable of producing patterns because the technology required to test these theories was not available; a problem which led to the dormancy of Turing's original ideas for many years. However, continuing advances in methodologies and technologies now allow future researchers to unbiasedly examine periodic pattern formation and be open to incorporating elements from other patterning modalities. This process has already begun [89], as there are now clear experimental examples of RD systems incorporating positional information such as the colouration and patterning of avian feathers [47,64] and the arrangement of reptilian ectodermal appendages [53,56] (Figure 4), all of which rely on defined signals from the somites. Indeed, in the developing limb, fingerprint patterns are also influenced by the shape of the developing digit and localised mesenchymal signalling centres [9,58], whereas the patterning of the bones and joints of the hand likely involves a complex interplay between multiple Turing systems and local mechanical forces.

Evaluating the roles of signal-based and MC patterning mechanisms in an unknown system can be challenging. A hallmark of RD systems is the presence of a pre-pattern that precedes any changes in cellular rearrangement, as seen for hair follicles and fingerprints [6,9]. However, the detection of a pre-pattern, especially in new systems is challenging. The continuing advancement of single cell and spatial RNA sequencing technology will aid identification of pre-pattern candidate markers through developmental trajectory analysis, and localisation of gene expression if enough temporal resolution is available. In hybrid or MC systems, whilst molecular pre-patterns are absent, local differences in cell density, proliferation rate, ECM alignment or increased mechanical strain can serve as symmetry-breaking events, and therefore should also be comprehensively examined when exploring new patterning systems.

Experimentally validating patterning mechanisms is challenging due to difficulties assessing parameters like molecular kinetics in biological tissues. Recent technical advancements such as fluorescence correlation spectroscopy for *in vivo* measurement of diffusion coefficients and ligand interactions/degradation of morphogens [90–92], as well as atomic force microscopy for assessing mechanical properties of tissues undergoing patterning [9,93], have enabled quantification of these parameters, and thus a more accurate dissection of the underlying patterning processes. Furthermore, mathematical models incorporating factors such as tissue growth [94], mechano-chemical feedback loops [95], and diffusiophoresis [96] into classical Turing RD systems, continue to be developed in an effort to more accurately reflect biological patterns.

Investigating pattern formation in non-mouse vertebrate models, such as zebrafish and chick embryos, offers numerous advantages including the ability to conduct live cell imaging and perform tissue manipulations during embryonic development. Ongoing advancements in transgenic technologies and reporter systems [97–99] make these organisms excellent models to study embryonic patterning events moving forward. Additionally, exploring pattern formation in more diverse species, such as reptiles [53,57] and wild rodents [100,101], will enable the study of how periodic patterning processes have evolved across taxa, potentially revealing novel patterning mechanisms.

The coexistence of RD and MC patterning modes in one tissue is likely more extensive than in hair follicles [6]. Indeed, Turing himself described the state of the system as having two parts, mechanical and chemical [13], making it highly likely that most periodic patterns utilise aspects of both RD and MC processes. The ability to dissect, decouple, and unbiasedly assess the influence and interaction of these distinct patterning modes on periodic pattern formation will, therefore, be essential for future studies.

Going forward, *in vitro* organoid and embryoid systems will be invaluable resources to complement *in vivo* experiments as the mechanical and signalling conditions can be rigorously controlled and manipulated, and they provide a system to study patterning events in developing human tissues. Potential systems include lung bud tip progenitor organoids generated from human pluripotent stem cells [102] which undergo bifurcation events that recapitulate the patterned branching structure of human lungs [103], and mammalian skin organoids [104,105] which develop periodically spaced hair follicles *in vitro*.

The exploration of other inputs into periodic pattern systems such as propagation of long-range signalling by calcium waves, differences in the metabolic properties of cells, and protein stability will also become accessible with the ever-advancing imaging, experimental, and modelling techniques. Ultimately, it will be up to ourselves as researchers to be rigorous in our assessment of patterning modality and be open to all avenues. It is only by understanding the complexities and interplay between RD and MC systems that we will be able to fully decipher how these beautiful arrangements form and leverage this knowledge to gain a greater insight into associated developmental disorders and inform future tissue regeneration strategies.

## Perspectives

- Determining how embryonic periodic patterns form is essential to fully understand how functional organs and tissues are generated, and why these processes are disrupted in certain congenital disorders. It is only by investigating how these patterns naturally form that we will to able to generate tissues *in vitro* that faithfully recapitulate the situation *in vivo*.
- Embryonic periodic pattern formation is more intricate than originally hypothesised and often involves a complex interplay between the MC and molecular signalling environment, coupled with further influences from neighbouring embryonic structures and tissue architecture.
- Going forward it will be essential to interrogate, and decouple, the roles of the MC and molecular environment in order for us to unbiasedly assess the patterning modalities of a system. Continuing theoretical and technological advances will enable this, particularly the development of new transgenic tools, organoid systems, and super-resolution microscopy approaches that allow us to experimentally validate more complex mathematical hypotheses and move us closer to delineating how different biological patterns form. Finally, the study of periodic patterns across diverse species has the power to not only provide insight into how these processes have evolved, but because it may reveal novel patterning modalities that have not yet been discovered.

## Open Access

Open access for this article was enabled by the participation of the University of Edinburgh in an all-inclusive *Read & Publish* agreement with Portland Press and the Biochemical Society under a transformative agreement with JISC.

## Abbreviations

ECM: extracellular matrix
MC: mechanocellular
RD: reaction–diffusion
